# Gut microbiota and cardiac arrhythmia: a pharmacokinetic scope

**DOI:** 10.1186/s43044-022-00325-2

**Published:** 2022-12-30

**Authors:** Omnia Azmy Nabeh

**Affiliations:** Department of Medical Pharmacology, Kasr Alainy Faculty of Medicine, Cairo University, Cairo, Egypt

**Keywords:** Gut microbiota, Gut dysbiosis, Pharmacokinetics, Arrhythmia, Anti-arrhythmic drugs, Anti-thrombotics

## Abstract

**Background:**

Dealing with cardiac arrhythmia is a difficult challenge. Choosing between different anti-arrhythmic drugs (AADs) while being cautious about the pro-arrhythmic characteristics of some of these drugs and their diverse interaction with other drugs is a real obstacle.

**Main body:**

Gut microbiota (GM), in our bodies, are now being considered as a hidden organ which can regulate our immune system, digest complex food, and secrete bioactive compounds. Yet, GM are encountered in the pathophysiology of arrhythmia and can affect the pharmacokinetics of AADs, as well as some anti-thrombotics, resulting in altering their bioavailability, therapeutic function and may predispose to some of their unpleasant adverse effects.

**Conclusions:**

Knowledge of the exact role of GM in the pharmacokinetics of these drugs is now essential for better understanding of the art of arrhythmia management. Also, it will help deciding when to consider probiotics as an adjunctive therapy while treating arrhythmia. This should be discovered in the near future.

## Background

Our gut is inhabited with many microorganisms that were reported to be crucial in determining our health and disease statuses. Gut microbiota (GM) are now considered as our hidden organ, being responsible for many physiological functions such as the development and regulation of our immune system, digesting complex polysaccharides and proteins, synthesis of: short chain fatty acids (SCFA), neurohormones (e.g., norepinephrine), and neurotransmitters [e.g., dopamine, serotonin, gamma aminobutyric acid (GABA)] [1]. On the other hand, although the pathophysiology of cardiac arrhythmia has been studied for decades, new gained concepts demonstrated the causal relationship between gut microbiota (GM) and cardiac arrhythmia [2]. Yet, the precise role of GM is not fully discovered. However, the emerging evidence about the impact of GM on drug metabolism will direct the future investigations for the importance of GM mapping to adjust the management of cardiac arrhythmia accordingly in the view of the pharmacokinetic impact of GM. This review provides an overview about the GM and their physiological function and discusses the contribution of GM in the pathogenesis of cardiac arrhythmia. Then, it summarizes the role of GM in drugs metabolism and how GM may alter the pharmacokinetics of drugs used in the management of arrhythmia, i.e., anti-arrhythmic drugs and anti-thrombotics. Finally, it outlines the gap between the current knowledge and the unsolved inquires.

## Overview on the impact of GM on human body

Human gut contains millions of microorganisms (bacteria, viruses, protozoa, etc.) that can be either: opportunistic, pathogenic or commensals and can be named collectively as gut microbiota (GM) [3]. Upon the different types of gut bacteria, Bacteroidetes and Firmicutes, together form around 90% of GM, while in the second place comes Verrucomicrobia and Actinobacteria. The balance between these bacterial phylae is important to maintain a healthy functioning ecological system. The gut, in turns, is composed of different types of cells; each possesses a certain function: 1. Enterocytes are cells responsible for the digestion and absorption of food and form a passive barrier against the passage of gut microorganism to the systemic circulation, 2. enteroendocrine cells with a secretory function secreting ghrelin, glucagon-like peptides, cholecystokinin, pancreatic polypeptide, and peptide YY, 3. goblet cells which secrete mucin forming a defensive blanket against any invading microorganisms, 4. Paneth cells which secrete anti-microbial peptides and immunomodulating proteins, 5. intestinal stem cells, and 6. immune cells [4, 5].

As regards the physiological function of GM, these bacteria are capable of producing many hormones, chemical transmitters, and bioactive compounds. Of these substances, dopamine, serotonin, noradrenaline, gamma-aminobutyric acid (GABA), choline, orexin, leptin, neuropeptide-Y are important GM metabolites that affect our psychological behavior, mood, appetite, and sleeping pattern [1]. Other GM metabolites are trimethylamine (TMA), indoxyl sulfate, and unconjugated and secondary bile acids that influence the host immune response [2].

GM can also digest complex dietary carbohydrates that our body cannot digest to secrete short chain fatty acids (SCFAs). Butyrate, acetate, and propionate are three chief SCFAs produced by GM. Besides being a source of energy, SCFAs bind to G-protein coupled receptors (GPCRs) to regulate satiety, insulin secretion and sensitivity, sympathetic activation and noradrenaline release [6]. Butyrate, and to a lesser extent propionate and acetate, are histone deacetylase (HDAC) inhibitors. By preserving the acetyl group, they can facilitate gene transcription of anti-microbial, anti-inflammatory, and anti-cancer peptides. These peptides are now being challenged in robust researches as disease modifying or therapeutic options. This was supported by several in vitro and in vivo studies that showed a positive impact of these three SCFAs on decreasing: the activation of nuclear factor-kappa ligand B (NF-*k*B) and the production of interleukin (IL)-I*β*, IL-6, IL-8, IL-10, IL-12, tumor necrosis factor-alpha (TNF*α*), and nitric oxide synthase (NOS) [7]. Furthermore, the SCFAs-mediated HDAC inhibition adjusts regulatory T cells (Treg) proliferation and function to avoid overwhelmed immune reactivity [8]. Moreover, human dendritic cells (DC) and toll-like receptors (TLRs), together with GM, play a principal role in the development and maturation of the host innate and adaptive immunity systems [9].

Meanwhile, the imbalance between the normal human–GM relationship is referred to as “dysbiosis.” This abnormal interrelation has a strong impact on the development and progression of many intestinal and extra-intestinal pathologies such as inflammatory bowel disease, allergy, metabolic syndrome, auto-immune diseases, and neuro-psychiatric illness [10]. When microbiota reach the systemic circulation, they start a vigorous inflammatory phenotype that are believed to be incorporated in many organs dysfunction. Probiotics (active beneficial bacteria), prebiotics (food promoting growth of microbiota), and synbiotics (mixed probiotics and prebiotics) are now used and being investigated as a therapeutic option in vast array of diseases [11].

## Role of GM in pathophysiology of arrhythmia

**Fig. 1:**
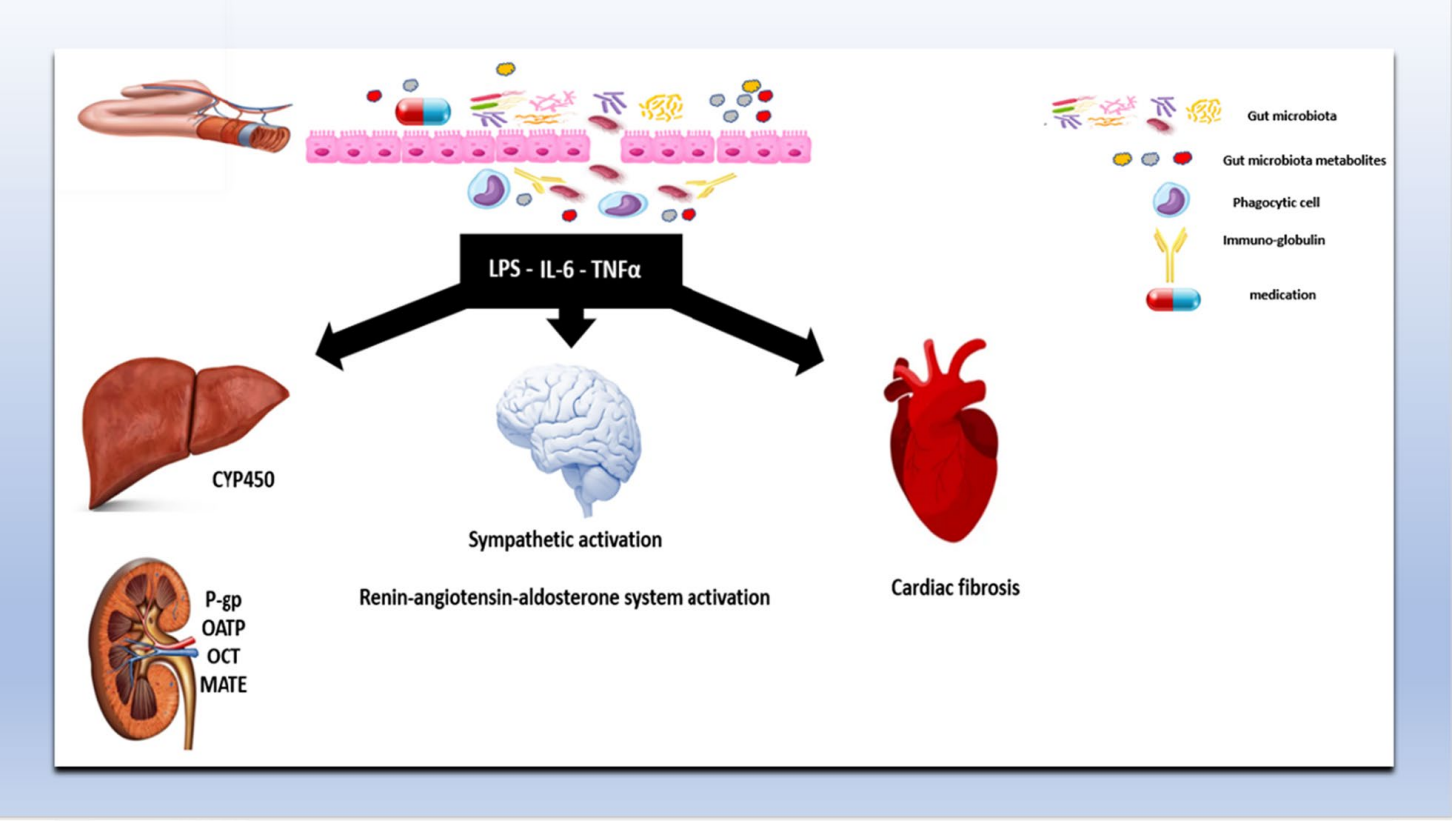
The role of gut microbiota in: the pathogenesis of cardiac arrhythmia and the pharmacokinetics of drugs used in the management of arrhythmia. *IL* interleukin, *LPS* lipopolysaccharide, *TNFα* tumor necrosis factor alpha

Apart from being sharing some risk factors such as heart failure, hypertension, obesity, and diabetes, many observations hypothesized that GM and arrhythmia are linked to each other. GM are speculated to predispose to cardiac arrhythmia by influencing autonomic sympathetic activity, Ca^2+^ handling, and structural remodeling [2] (Fig. 1).

The bacterial lipopolysaccharide (LPS) is considered an endotoxin which upon reaching the host blood, it increases the release of pro-inflammatory cytokines that was observed to decrease the expression of L-type Ca^2+^ channels and shorten effective refractory period (ERP) [12]. LPS can also indirectly, through mediating atherosclerosis and heart failure, increase the risk of cardiac arrhythmia especially atrial fibrillation (AF) [13].

Local injection of the GM-TMA oxidation product (TMAO) was noticed to activate the atrial autonomic fibers promoting arrhythmia. Furthermore, some studies reported higher levels of TMAO in patients with atrial fibrillation (AF) [14] along with increased risk of thromboembolic events [15]. TMAO was also reported to induce cardiomyocyte hypertrophy and fibrosis in rats and in vitro ventricular cardiomyocytes. In addition, TMAO has a pro-atherosclerotic activity and may promote aortic stiffness, metabolic syndrome, and renin-angiotensin system regulation predisposing to arrhythmogenesis [16, 17].

Furthermore, the GM metabolite, choline showed a weak muscarinic receptor agonistic effect that seems to activate acetylcholine-dependent potassium channels at high concentrations, thereby shortening ERP [18].

Similarly, indoxyl sulfate that is derived from the metabolic action of GM on dietary tryptophan, in a concentration > 0.65 µg/mL, was documented to potentiate arrhythmogenesis through disturbing calcium handling by cardiomyocytes and enhancing after depolarizations [19, 20]. Interestingly, AF catheter ablation reduced indoxyl sulfate concentration [21].

However, the association between the aforementioned GM metabolites and arrhythmia remains to be a rich area of investigation as regards the concentrations of these different arrhythmogenic GM metabolites that can mediate clinical arrhythmia, as well as their cellular and molecular pathways.

## Role of GM in drug metabolism

It is well-known that the pharmacokinetic (PK) properties of available drugs differ according to many variables, e.g., chemical structure, lipid solubility, prodrugs versus active drugs, drugs with enteric-release formulae, site of absorption, gastrointestinal pH, way of metabolism, route of elimination, and drugs substrate for enterohepatic circulation. Drug metabolism per se consists of 3 phases. Phase I includes oxidation, hydrolysis and reduction, phase II involves drug conjugation, and phase III refers to the drug transport system [22]. Cytochrome P 450 (CYP 450) enzymes, cellular (cyto), heme pigment-containing (chrome P), enzymes that absorb light at a wavelength 450 nm, are present predominantly in human liver cells and to a lesser extent in the intestine, kidneys, lungs, and placenta as well as in some plants and microorganisms. These enzymes are divided into eighteen families and forty-four subfamilies, with six enzymes, CYP1A2, CYP2C9, CYP2C19, CYP2D6, CYP3A4, and CYP3A5 being responsible for more than 90% of the metabolizing capacity of the CYP450 enzymes [23]. These enzymes through catalyzing S-oxidation, epoxidation, O-dealkylation, and hydroxylation are capable of bioactivating, metabolizing, and detoxifying drugs and other xenobiotics. They are also responsible for fatty acids and steroids metabolism that result in some hormones synthesis and breakdown [24].

It was first believed that only colonic microbiota can affect drugs pharmacokinetics (PK). However, new evidence tends to include small intestinal microbiota as well [25]. An average of 40 drugs have been studied as regards the effect of GM on their metabolism [26]. GM was identified to modulate drug metabolism of these drugs in different ways. They can directly secrete drug-metabolizing enzymes, adjust host metabolizing enzymes expression and action, and the bacterial metabolites can compete with drugs for receptors and transporters [27]. Interestingly, GM was found to possess CYP-like enzymes that are also capable of catalyzing phase I and II reactions of drug metabolism [28]. Also, GM has other active enzymes, i.e., β-glucosidase, β-glucuronidase, aryl-transferase, nitro-reductase, nitrate reductase, and azo reductase [29]. So that, the dynamic activity of GM can affect some of the PK steps, namely prodrugs activation, drug metabolism (especially for drugs which undergo enterohepatic circulation) and drug elimination. These alterations may cause low inefficient drug plasma level or predispose to drug toxicity or unpleasant adverse effects.

As regards prodrug activation, it is well known that sulfasalazine, a prodrug used in treatment of ulcerative colitis, can only be converted to its two active components, sulfapyridine and 5-aminosalicylic acid after being liberated by the microbial azo-reductase enzymes in the intestine. This finding was supported by Mikov et al. [30] who found a significant increase in sulfasalazine reduction following probiotic treatment. Moreover, lovastatin is hydrolyzed to its active lipid-lowering *β*-hydroxy acid metabolite by GM that potentiate its pharmacological effect. GM can also release the active sulfanilamide from sulfa antibiotic. In the same context, the activation of the prodrug prontosil to active sulfanilamide was inhibited by the coadministration of antibiotics that attenuated GM growth and metabolizing activity [31].

With respect to drug metabolism, many examples are present signifying the role of GM in this pivotal process. Tacrolimus, a calcineurin inhibitor immune-suppressant drug, displays a narrow therapeutic index which necessitates adequate drug monitoring. Lee et al. [32] indicated the effect of GM on tacrolimus PK that was furtherly supported by Scott et al. [33] who found that patients with abundant Faecalibacterium often require higher doses of tacrolimus. Another example for the role of GM on drugs metabolism is patients harboring high levels of the GM metabolite, p-Cresol who were found to be more liable to acetaminophen-induced hepatotoxicity as p-Cresol competes with acetaminophen for hepatic sulfonation during phase II of drug metabolism [34]. Furthermore, GM can also deactivate some drugs by increasing their metabolism, e.g., levodopa [35], calcitonin [36] and digoxin [37].

Preclinical studies have also demonstrated that chronic antibiotic usage can alter the therapeutic activity of some drugs through affecting the GM-mediated drug handling. Antibiotics also could improve the safety profile of some drugs, for example, the deleterious effects of olanzapine, an antipsychotic medication known for predisposing to metabolic disorder, were attenuated with antibiotics co-treatment [38].

Nevertheless, the immune-regulating potential of GM mediated some anti-cancer therapeutic functions, specifically anti-CTLA-4 and anti-PD-L1 targeting drugs that showed better outcomes in patients with some species of Bacteroides and Bifidobacterium, respectively. These actions are hypothesized to be related to the action of GM on DCs which are essential to trigger proper T-cells response [39].

Another mechanism by which GM can induce alteration in drug PK is “gut dysbiosis.” Gut dysbiosis and loss of intestinal tight junctions’ integrity can merely facilitate the passive passage of drugs to systemic blood and then disturb their oral bioavailability. Moreover, the recognition of the bacterial LPS, by host immune system and the resulting systemic inflammation can cause significant alteration in the expression of CYP450 enzymes. In general, inflammatory cytokines released during inflammation, downregulate the expression and function of CYP enzymes. Animal studies have found an inverse relation between LPS and CYP1A1, CYP2E1, CYP3A11, CYP4F4, and CYP4F5 activity [40]. In contrast, CYP 4A2, 4A1, and 4A3 were found to be elevated in rats treated with LPS [41].

Not surprisingly, chronic kidney disease (CKD) affects GM ecosystem, with GM and dysbiosis being able to contribute to renal inflammation which will be reflected on renal-drug handling. The digestion of proteins produces *α*-amino nitrogen that are normally excreted in feces. The overproduction of *α*-amino nitrogen (due to either excessive protein intake or decreased dietary fibers) will be then converted by GM into uremic toxins. The passage of these toxins to the kidneys after losing the integrity of gut barrier contributes to renal affection that will attenuate the drug-handling capacity of the kidneys [42]. In a study that included 30 patients with end-stage renal disease (ESRD), the gut bacterial DNA was detected in the blood of 20% of patients with higher levels of C-reactive protein (CRP) and IL-6 than detected in the remaining studied patients. This supports the speculation that the translocated gut bacteria contribute to the pathology of CKD [43]. Furthermore, the GM metabolites, p-cresol and indoxyl sulfate levels increase dramatically in CKD and gut dysbiosis, where both metabolites have albumin binding affinity and can attenuate some drugs activity and clearance [44]. ESRD was also associated with decreased SCFA-producing bacteria growth which included Lactobacillaceae and Prevotellaceae who are known for producing butyrate. The latter plays a crucial role in maintain intestinal barrier function and reduce inflammation. Furthermore, in an interesting study from Japan, Kuno et al. [45] found a significant relation between gut dysbiosis and the expression of some drugs metabolizing enzymes and transporters in both, kidney and liver. In germ-free (GF) mice, Kuno found a significant reduction in organic anion transporter polypeptide (OATP)1a1 expression, while sulfotransferase (Sult)1a1 was increased which may be reflected on some drugs PK.

These examples and many others will direct future researches to give more attention to the central role played by GM in the PKs of available drugs.

## The impact of GM on drugs used in management of cardiac arrhythmia

Anti-arrhythmic drugs (AAD) are grouped in the Vaughan Williams classification into 4 groups according to the ion channel or receptor they exert their anti-arrhythmic effect on. Class I blocks Na + channels, Class II blocks the sympathetic activation of Beta-2 (*β*2) adrenergic receptors on the heart, Class III blocks mainly K + channels, and Class IV are calcium channel blockers. Class I is furtherly divided into 3 subgroups (Ia, Ib, Ic) according to the state of the Na + channels they prefer to act upon. Class I and class III anti-arrhythmic drugs are known to have a pro-arrhythmic potential that may itself aggravate arrhythmia (Vaughan [46]).

Some anti-arrhythmic drugs are metabolized to active metabolites. These drugs are: Amiodarone (N-desethyl-amiodarone), propafenone (5-OH propafenone), lidocaine (glycylxylidide and mono-ethyl glycylxylidide), verapamil (nor-verapamil), Quinidine (3-OH quinidine), disopyramide (mono-N-dealkyl disopyramide). Some AADs also exert a nonlinear PKs (lidocaine, disopyramide, amiodarone, propafenone, and verapamil). These active metabolites can be useful for their therapeutic effect and may also predispose to drug toxicity. Furthermore, genetic polymorphism affecting AADs metabolism and drug–drug interactions are all factors that can influence the PK properties of the AADs [47, 48].

According to route of elimination, AADs can be distinguished into 3 groups: group 1. undergoing extensive hepatic metabolism (e.g., lidocaine, propafenone, amiodarone, mexiletine, and verapamil), group 2. renal elimination with more than 50% of the drug are excreted unchanged in urine (e.g., disopyramide and sotalol), and group 3. mixed hepatic and renal elimination (e.g., procainamide, flecainide, quinidine, and tocainide). Because of the first pass metabolism, most of group 1 drugs show a highly variable oral-bioavailability that can be furtherly disturbed with any reduction of hepatic blood flow or hepatic insufficiency. In contrast, group 2 and group 3 show a nearly constant bioavailability, with the PK of group 2 being correlated with creatinine clearance [48–51].

Although the individualized role of GM in AADs metabolism is not fully recognized, some information could be proposed from the chemical structure and the pharmacokinetics properties of the AADs (shown in Table 1).

**Table 1:** Pharmacokinetic properties of some anti-arrhythmic and antithrombotic drugs commonly used in management of cardiac arrhythmia

			Major route of elimination	Involved cytochrome P450 enzymes	Protein binding %	Transporters	References
Anti-arrhythmic Drugs	Class Ia	Quinidine	Hepatic and renal	CYP3A4, CYP2D6	80	P-glycoprotein, OCT2, OCT3	[47, 52, 53]
		Disopyramide	Renal	CYP3A4	50–65	OCT2, OCT3	[53]
		Procainamide	Hepatic and renal	CYP2D6	20	MATE; OCT3	[53–55]
	Class Ib	Lidocaine	Hepatic	CYP1A2, CYP3A4	60–80	OCT3	[53, 55, 56]
		Phenytoin	Hepatic	CYP2C19; CYP2C9	90	OCT3	[53, 57]
		Mexiletine	Hepatic	CYP2D6	70	OCT2, OCT3	[53, 58, 59]
		Tocainide	Hepatic and renal	CYP3A4	50		[60]
	Class Ic	Flecainide	Hepatic and renal	CYP2D6	20	OCT2	[59, 61]
		Propafenone	Hepatic	CYO2D6, CYP3A4	90	OCT2	[59, 62]
	Class II	Esmolol	Blood esterase	…….	55		[63]
		Metoprolol	Hepatic	CYP2D6			[64]
	Class III	Amiodarone	Hepatic	CYP1A2, CYP2C9, CYP2D6	96	P-glycoprotein, OAT2B1	[47, 65]
		Sotalol	Renal	CYP3A4	0	OCT2	[47, 65, 66]
		Ibutilide	Hepatic and renal	undetermined	…		[67]
		Dofetilide	Hepatic and renal	CYP3A4	60–70	MATE: OCT2	[55, 68]
	Class IV	Verapamil	Hepatic	CYP3AF, CYP3A5	90	P-glycoprotein, OCT2	[47, 52]
		Diltiazem	Hepatic	CYP3AF, CYP3A5	70–80	P-glycoprotein	[47, 52]
	Others	Adenosine	Cellular metabolism	……	……		[69]
		Digoxin	Renal	……	20–30	P-glycoprotein	[47, 70]
Antithrombotic		Warfarin	Hepatic	CYP1A2, CYP2C19, CYP3A4	99		[71]
		Aspirin*	Hepatic	CYP2D9	58.3–81.7#	OAT1	[72–74]
		Dabigatran	Renal	CYP3A4	35	P-glycoprotein	[70, 75]
		Edoxaban	Renal	….	40–59	P-glycoprotein	[70, 76]
		Rivaroxaban	Hepatic and renal	CYP3A4, CYP3A5, CYP2J2	92–95	P-glycoprotein	[70, 77]
		Apixaban	Hepatic and renal	CYP3A4, CYP1A2, 2C8, 2C9, 2C19	93	P-glycoprotein	[47, 70, 78]

*Other Aspirin metabolizing enzymes: *N*-acetyl transferase2(NAT2) an UDP-glucuronosyltransferase 1A6(UGT1A6). # The plasma protein binding of aspirin ranges from approximately 58.3 for aspirin and 81.7 for salicylates. *MATE* multi-drug and toxin extrusion proteins, *OAT* organic anion transporter, *OCT* organic cation transporter

### Protein-binding

As mentioned before, the GM metabolites, p-Cresol and indoxyl sulfate exhibit high protein binding capacity, are excreted by kidneys via tubular secretion, and accumulate with failing kidneys [79]. So that, they may compete with some AADs that bind in considerable amount to plasma proteins and/or dependent on tubular secretion, e.g., disopyramide which has a binding affinity to the plasma protein, plasma acid alpha-1-glycoprotein and will be reflected on its renal clearance [80].

### Transporters

Recently, few studies have reported the effects of probiotics on some drug transporters in the gut. Lactobacillus acidophilus and Lactobacillus rhamnosus were demonstrated to upregulate P-glycoprotein (P-gp) in the intestine [81]. This suggests that absorption and elimination of anti-arrhythmic and anticoagulants substrates to P-gp can be attenuated by probiotics. In addition to the role of GM on OATP1a1 and Sult1a1 [45] and OATP2B1 [82].

### Amiodarone

The GM, Escherichia coli Nissle 1917 was identified to increase the bioavailability of amiodarone secondary to a reduction in the gut pH, enhancing the drug ionization and absorption. It is also theorized that the increased bioavailability of amiodarone is due to the upregulation of the OATP2B1 transported gene by the GM. Furthermore, the amiodarone metabolite, N-desethylamiodarone concentration was increased 1.5-fold after administration of the probiotic *E. coli* Nissle 1917 [82]. Owing to its narrow therapeutic range, certain gut bacteria, in certain conditions, can precipitate amiodarone-mediated toxicity.

### Digoxin

Some strains of the anaerobic gut bacteria, Eggerthella lenta, were demonstrated to be able to inactivate digoxin owing to their cardiac glycoside reductase (cgr) operon. So, in patients with abundant Eggerthella lenta-cgr positive strains, antibiotics may be needed to increase serum digoxin level [37]. Moreover, being substrate to P-gp transporters, digoxin level can be attenuated by GM as previously discussed [70, 81].

### Metoprolol

In contrast, the β1-adrenergic blocker, metoprolol, was observed to prevent bacterial overgrowth in the gut and the subsequent bacterial translocation. This action was shown to be secondary to the stimulation of the gut motor function [83]. Therefore, the chronic use of metoprolol may itself prevent dysbiosis. However, the impact of metoprolol on GM needs further evaluation.

### Aspirin

Low-dose aspirin is used to prevent thromboembolic events in some arrhythmic settings. Kim et al. [84] found that oral and not intravenous, ampicillin-pretreated rats showed prolonged bleeding time after treatment with aspirin in comparison to control group [85]. This alteration in aspirin antithrombotic effect is speculated to result from decreased carboxylesterase activity in the gut by oral ampicillin, reducing some enterococci, enterobacteria, and lactobacilli strains, decreasing the pre-systemic conversion of aspirin to salicylic acid, and increasing its absorption and systemic effects [29, 85].

### Warfarin

It is well known that vitamin K can antagonize the anticoagulant activity of warfarin. Interestingly, some GM was identified as vitamin K producers, e.g., Bacteroides spp, Enterobacter agglomerans, Staphylococcus capitis, and Enterococcus faecium [86]. This supports the potential of some microbiota strains to antagonize vitamin K antagonist, warfarin.

In the view of the above-mentioned information, GM forms a real obstacle while considering AADs and anti-thrombotics in the management of cardiac arrhythmia. But whether this interruption in the drugs PKs by the action of GM is of clinical effect or not, it will need more deep and detailed investigations with special attention to the specific strain and the strain concentration that is of important clinical impact.

## Arrhythmia and dysbiosis co-morbidities

Low cardiac output disturbs the normal physiological function of many organs. Of these organs, is the gut. When the epithelial barrier and tight junctions are lost,  this will facilitate the translocation of the gut microbes to the blood, predisposing to cardiac arrhythmogenesis and disturbing drug metabolism and activity as previously explained. Low cardiac output may result from a disease that itself share or cause cardiac arrhythmia, e.g., heart failure [84], valvular dysfunction [87], and pulmonary hypertension [88].

In addition, elderly population is characterized by being at higher risk of arrhythmia and gut dysbiosis. Evidences for loss of intestinal barrier function, a decrease in the saccharolytic bacteria that is matched with an expansion in the proteolytic bacterial growth, and a significant rise in plasma inflammatory markers promoting a chronic inflammation are more common in elderly [88] which may result in an unfavorable response in management of cardia arrhythmia.

The interaction between drugs and the impact of these drugs on GM may also result in uncontrolled arrhythmia [29, 70]. The influence of antibiotics on drugs metabolism and the GM is another indirect cause of uncontrolled arrhythmia.

So, many factors should be taken in consideration while evaluating any case of uncontrolled arrhythmia to exclude any possible neglected insult to the GM, being widely affected and interacting with some causes of arrhythmia and the arrhythmia drug controllers, anti-arrhythmic and anti-thrombotics as well.

## Conclusions

GM have an intimate symbiotic relationship with human bodies. However, gut dysbiosis and the enzymatic activity of some gut-bacterial strains are believed to alter the PK properties of many drugs. Anti-arrhythmic drugs substrate to CYP-mediated metabolism is at higher risk to lose their therapeutic effect and/or safety profile with any disturbance of GM balance. GM can also disturb some anti-arrhythmic drug absorption and elimination as well as attenuating some antithrombotic drugs activity. Enrolling the possible interfering role of GM in the PK of drugs used in management of cardiac arrhythmia will help to better understand the variable/unexpected inter-individual drug responses and the potential of probiotics to help passing-over this noisy challenge.

## Data Availability

Not applicable.

## Abbreviations

AADs: Anti-arrhythmic drugs
cgr: Cardiac glycoside reductase
CKD: Chronic kidney disease
CRP: C-reactive protein
CYP: Cytochrome
ERP: Effective refractory period
ESRD: End-stage renal disease
GM: Gut microbiota
GPCR: G-protein coupled receptor
DC: Dendritic cells
IL: Interleukin
LPS: Lipopolysaccharide
MATE: Multi-drug and toxin extrusion protein
NF-*k*B: Nuclear factor- kappa ligand B
NOS: Nitric oxide synthase
OATP: Organic anion transporter polypeptide
P-gp: P-glycoprotein
PK: Pharmacokinetic
SCFA: Short-chain fatty acid
Sult: Sulfotransferase
TLRs: Toll-like receptors
TMA: Trimethylamine
TMAO: Trimethylamine oxidation metabolite
TNFα: Tumor necrosis factor-alpha
